# Effects of a moderate-to-high intensity resistance circuit training on fat mass, functional capacity, muscular strength, and quality of life in elderly: A randomized controlled trial

**DOI:** 10.1038/s41598-019-44329-6

**Published:** 2019-05-24

**Authors:** Pablo Jorge Marcos-Pardo, Francisco Javier Orquin-Castrillón, Gemma María Gea-García, Ruperto Menayo-Antúnez, Noelia González-Gálvez, Rodrigo Gomes de Souza Vale, Alejandro Martínez-Rodríguez

**Affiliations:** 1 Grupo de Investigación en Salud, Actividad Física, Fitness y Comportamiento Motor (GISAFFCOM), Faculty of Sport, Catholic University San Antonio of Murcia (UCAM), Murcia, Spain; 2 Faculty of Sciences, University of Alicante, Alicante, Spain; 3 Faculty of Education, University of Extremadura, Cáceres, Spain; 4 Exercise Physiology Laboratory, Estácio de Sá University, Cabo Frio, Rio de Janeiro Brazil

**Keywords:** Skeletal muscle, Geriatrics

## Abstract

Physical exercise is considered an important intervention for promoting well-being and healthy aging. The objective was to determine the effects of moderate-to-high intensity resistance circuit training on different parameters of body composition, functional autonomy, muscular strength and quality of life in elderly. A randomized controlled trial was conducted. A total of 45 subjects (27 females, 18 males) aged between 65–75 years old from Murcia (Spain) were divided by sex, and randomly to experimental group (n = 33, mean age 69 ± 3.2 years old) receiving 12 weeks of moderate-to-high intensity resistance circuit training and control group (n = 33, mean age 70 ± 4.1 years old) receiving no exercise intervention. Intra-group comparison, the experimental group showed a significant increment of lean body mass in women and men, which also presented a decrease of fat mass. Both sex presented a significant improve in functional autonomy, and significately higher values of muscular strength. But no changes were observed regarding quality of life in these groups. The control group did not show any differences pre and post-intervention in women, but in men presented an increment of body mass index and total weight post-intervention. No changes were showed in the other variables. Similar results were founded at inter-group comparison. The moderate-to-high intensity resistance circuit training showed increase in total lean body mass, improvements in functional capacity and significantly increase in upper and lower muscular strength in women and men. Progressive resistance circuit training should be promoted for the elderly as it has the potential to improve physical performance, thereby prolonging healthy independent aging.

## Introduction

The world population has been experiencing significant ageing, this means that the process has resulted in rising proportions of older persons in the total population since the mid-twentieth century^1^. The proportion of population aged 65 years and over has risen from 11.2% in 1981 to 18.2% in 2013, and is expected that by 2050 more than one third of the total Spanish population^2^.

Maintaining the ability to work and earn a living, independence, and self-sufficiency in daily life and leisure time will therefore become increasingly important over the coming decades. A crucial factor in this is sustaining a high individual strength capacity^3,4^.

Variation in weight and body composition (BC), have important implications for the health and functional capacity of the elderly population^5,6^. Muscle mass declines with age and is gradually replaced by a fat mass^7^ Changes in BC associated with ageing may result in Body Mass Index (BMI) increase of 1.5–2.5 kg/m^2^ in both men and women, even when body weight remains constant^8–10^. Since the BMI of less than 25 kg/m^2^ can be responsible for frailty and higher mortality in the elderly, using of specific criteria for this population can be recommended^7^. Excessive fat mass is associated with risk factors such as elevated plasma cholesterol, plasma glucose, and resting blood pressure, which contribute to the development of type 2 diabetes and cardiovascular disease^11,12^. Furthermore, low muscle mass in elderly is related with physical inactivity and inadequate nutrient energy intake^13^. Resistance training (RT) is recommended in the management of obesity and metabolic disorders^12^.

The need for maintenance of physical activity (PA) throughout life is widely recommended by the scientific literature, in particular, during the stage at which aging accentuates the decline of the systems responsible for the functionality of the body, affecting the ability of the elderly to participate in daily activities, which consequently affects their functional autonomy (FA), thus increasing the risk of developing diseases with physical and psychological consequences^14,15^.

Aging develops in older people, as a reduction in muscle size, strength, and flexibility, associated with changes in fat mass, muscular mass, and cardiovascular diseases^16^. Muscular strength (MS) gradually decreases from the 30th year. The risk of acute problems owing to falls and injuries and chronic recurrent and degenerative illnesses rises^17,18^.

The American College of Sports Medicine (ACSM) Position Stand highlight the importance for older adults to do RT^19^.

To promote and maintain health and physical independence, older adults will benefit from performing activities that maintain or increase MS and endurance. Muscle-strengthening activities include a progressive RT program, that use the major muscle groups^20^. RT programs for older population are determinant to reduce the negative impact of physiological aging. Losses in muscle strength and motor development are factors related to functional disability and dependence^21^.

Moderate-intensity RT (≤60% RM) produce improvements in muscle strength and muscle power. Moreover, this kind of training cause significant adaptations in BC, decreasing body weight (−1.9%) and body mass index Folds (−2.6%) in populations older than 65 years^22–24^. Regarding High-intensity RT (≥75% RM) produces greater increases in strength and power output. High-intensity training programs (>75% of 1 RM) are related to improvements in muscle strength around 21–97% after an intervention of 10 to 52 weeks in adults over 65 years old^24–32^. For his part, Resistance Circuit Training (RCT) causes improvements in muscle strength, VO2 max, body composition and the time spend in performing daily activities in the elderly^22,33,34^.

Quality of life (QOL) is a factor directly linked to the context of aging, one of those responsible for the increase or decrease in the longevity of the population^1,35^. Given that aging, function and MS decline, represents an inevitable condition for an increasing number of elderly, the development of non-pharmacological strategy is important to maintaining health throughout adult life. PA, including RT, has both health promoting and disease prevention benefits. In order to maintain an independent lifestyle, an increase in PA of the elderly it is essential to preserve muscle mass and strength and would have the greatest impact on their health and QOL^19^.

The main purpose of the study was therefore to investigate the effects of 12 weeks of moderate-to-high intensity resistance circuit training (MHRCT) on BC, FA, MS and QOL on healthy elderly people. We hypothesised that MHRCT might reduce fat mass, BMI and improve lean body mass (LBM), FA, MS, and QOL perception in the elderly.

## Materials and Methods

### Participants and study design

**Figure 1:**
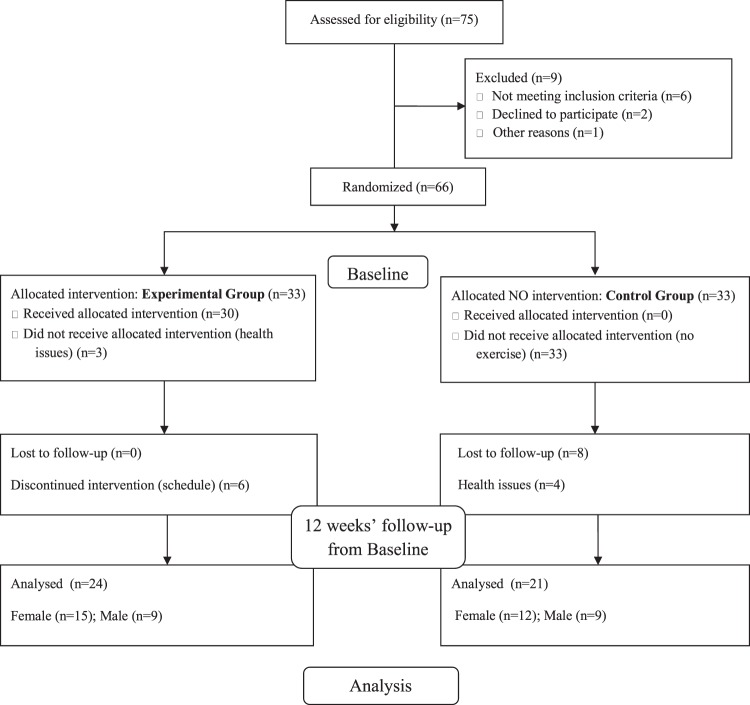
Flow diagram of the sample.

A total of 75 subjects were recruited from an elderly social groups from Murcia (intentionally selected) and voluntarily participated in the study. Participants who meet the inclusion criteria were electronically (https://www.randomizer.org) randomized by blocked-design into two arms: a control group (CG), and an experimental group (EG). After 9 subjects were excluded, 66 were randomly allocated to either the EG (n = 33, mean age 69 ± 3.2 years old) or CG (n = 33, mean age 70 ± 4.1 years old) at baseline. In addition, 21 more subject were excluded for the study (discontinued intervention or health issues). Subsequently, a total of 45 subjects (27 females, 18 males), 24 subjects in the EG and 21 subjects in the CG completed the 12 weeks’follow-up assessments. This procedure was established according to “CONSORT” statement (http://www.consort-statement.org), as displayed in the flowchart in Fig. 1.

The general characteristics of the subjects are given in Table 1. All subjects were originally from Murcia, Spain and were chosen according the following inclusion criteria: age ≥65 years, they never attended classes of fitness academies or following PA, and had no experience with ST. Exclusion criteria included any history of neuromuscular, metabolic, hormonal, cardiovascular diseases. Subjects were not taking any medication that could influence hormonal and neuromuscular metabolism.

**Table 1:** Descriptive statistics and inter-group analysis.

Body composition	Women (n = 27)					Men (n = 18)				
	Control (n = 12)		Experimental (n = 15)		Baseline comparison	Control (n = 9)		Experimental (n = 9)		Baseline comparison
	X	SD	X	SD	p Value	X	SD	X	SD	p Value
Weight (kg)	71.08	12.29	71.63	10.05	0.896	80.75	15.12	85.63	14.42	0.506
BMI (kg/m^2^)	26.09	3.34	27.57	4.43	0.142	26.67	4.51	27.97	3.74	0.216
Fat mass (%)	30.85	5.67	36.41	6.58	0.057	30.31	6,89	27.00	5.34	0.282
LBM (kg)	49.43	11.52	45.21	5.53	0.209	59.35	11.13	62.06	8.26	0.575
**GDLAM**										
W10m	5.66	0.59	5.56	1.08	0.770	5.26	0.36	5.18	0.96	0.818
SSP	9.26	1.27	7.08	1.78	0.001	11.24	1.24	7.64	1.15	0.001
SPP	3.57	0.71	3.97	1.45	0.388	3.00	0.63	3.39	2.10	0.622
SCMA	35.57	6.16	38.45	6.26	0.236	36.76	5.84	37.66	5.44	0.749
PTS	13.69	5.59	12.84	3.44	0.624	18.64	6.57	16.49	4.43	0.437
GI	20.36	3.88	19.94	3.82	0.685	23.38	4.60	21.71	3.99	0.435
**Strength conditioning**										
1 RM CHP (kg)	25.62	6.05	11.01	3.39	0.001	28.93	6.41	21.91	6.33	0.039
1 RM MP (kg)	7.86	4.08	5.17	3.38	0.068	10.66	3.79	12.96	7.91	0.468
1RM LE - (kg)	31.86	3.29	30.43	9.73	0.631	29.40	3.90	49.19	15.32	0.003
1 RM HE(kg)	32.90	9.50	34.30	12.50	0.749	29.41	10.42	45.64	16.89	0.033
**Quality of Life**										
Facet 1	90.63	5.65	92.19	9.82	0.627	89.06	4.42	87.50	13.62	0.761
Facet 2	81.25	8.43	88.28	9.92	0.059	79.69	9.30	81.25	19.52	0.84
Facet 3	76.56	10.70	89.06	11.75	0.008	81.25	11.57	82.64	17.05	0.849
Facet 4	80.21	10.26	82.03	19.35	0.770	82.81	9.30	76.39	19.46	0.409
Facet 5	69.79	7.92	75.00	17.82	0.355	71.09	8.80	76.39	16.76	0.437
Facet 6	58.85	10.13	86.72	10.67	0.001	62.50	11.57	78.70	15.72	0.03
Global QOL	76.22	7.39	85.52	11.12	0.019	77.74	8.39	80.41	14.92	0.661

Data expressed as difference between the variable at the beginning and end of the study; M: Mean; SD: Standard Deviation; Mean dif: Mean difference; OP: Observed power; BMI: Body Mass Index; LBM: Lean Body Mass; GDLAM: Latin American Group for Maturity protocol; W10m: walking 10 m test; SSP: standing up from a seated position test; SPP: standing up from a prone position; SCMA: standing up from a chair and moving about the room; PTS: putting on and taking off a shirt; GI: GDLAM index; RM: 1-Repetitium maximum; CHP: Chest Press; MP: Military Press; LE: Leg Extension; HE: Hip Extension; QOL: Quality Of Life.

All measurements were applied using standardized protocols and were made by two of the investigators (P.J.M.P., F.J.O.C.). Data collection pre and post-training (12 weeks) was performed in two days. On the first visit, between 8:00 and 9:00, biological test and quality of life measures were made. On the second day (24 hours after), the submaximal strength test was applied. Participants were carefully informed about the possible risks and discomforts that could occur and were asked to complete a health history questionnaire and to sign a consent form. This study was performed in accordance with the standards of 1964 Declaration signed in Helsinki, and the protocol was approved by the Ethical Committee (CE031207) of the Catholic University of Murcia (UCAM), Spain and registered at the Clinical Trial Registry (NCT03551132).

#### Sample size and power

Rstudio 3.15.0 software was used to calculate the sample size. It was set at a significance level of α = 0.05. Assumed error was calculated according to the standard deviation established for leg extension test in previous studies^29^ and a significance level of α = 0.05. A total of 45 participant completed the intervention. It was provides a power of 95% with a assumed error of 0.11 kg.

#### Nutrition and dietetics

Isocaloric diets based on Mediterranean food were registred based on a 24 h food register (4 weekdays and 1 weekend day). Diets were analyzed and designed using Diet source software (Novartis, Barcelona, Spain), and were adapted accordingly to each particular subject. Diets showed the following distribution aproximately: 1 g of protein/kg of body weight (in according with recommendations)^36–38^, 25–30% of fat of total kcal and the remaining kcal was completed with carbohydrates as the main macronutrient, corresponding to 55–60% of total kcal. In general terms, the subjects performed 5 daily intakes.

### Measures

#### Body composition

Total body weight and % fat mass, and by defect, LBM was measured after removal of shoes and heavy outer clothing, using a Tanita BC-418 MA (Tanita Corporation, Arlington Heights, IL) to the nearest 0.1 kg. Standing height without shoes was measured using a Seca 202 stadiometer (Seca, Hamburg, Germany) to the nearest 0.1 cm. BMI was calculated as the ratio of weight to squared height^39^.

Analysis of body composition by bio-impedance requires a strict protocol hours before data collection^40^. Published suggestions, were followed to run the protocol used for bio-impedance assessment (pre and post intervention) for the subjects. All the patients were BMI between 16 and 34 kg/m^2^ without abnormal hydration. The protocol included: fasting condition of participants, assessed in the early morning time and evaluated only with the same underwear clothes without socks and shoes.

#### Functional autonomy

The Latin American Group for Maturity (GDLAM) protocol was used to evaluate functional autonomy^15,41–43^. It is composed of five tests: walking 10 m (W10m); standing up from a seated position (SSP); standing up from a prone position (SPP); standing up from a chair and moving about the room (SCMA); and putting on and taking off a shirt (PTS). These tests are used in a mathematical formula to calculate the GDLAM index (GI). The equipment used consisted of a 48-cm chair (measured from the seat to the floor), a stopwatch (Casio, Malaysia), two cones, a mat (Olive Fitness, Spain) and a sunny brand metal tape measure.

#### Muscular strength

The predicted 1-repetition maximum (1-RM) testing protocol followed the procedure previously described by Brzycki^44,45^. Upper body strength was measured by evaluating the strengths of the deltoids, triceps, and muscles by having the subjects perform a chest press (CHP) and military press (MP); lower body strength was measured by assessing the strengths of the gluteals, hamstrings, and quadriceps muscles by having them perform a leg extension (LE) and hip extension (HE). All machine based exercises were performed on Technogym equipment (Italy).

#### Perceived exertion

Ratings of perceived exertion were assessed using OMNI-RES scale of perceived exertion^46,47^ on an eleven-point scale (0 = extremely easy to 10 = extremely hard). Standard instructions for the OMNI-RES were read to the participants before each testing session. Previous evidence has supported the concurrent validity of this measure in performing upper and lower body resistance training programs^46,47^.

#### Quality of life questionnaire

Quality of Life in the elderly (WHOQOL-OLD)– a Spanish version: is an instrument developed by Power *et al*.^48^, translated and validated for the Spanish language^35^. The WHOQOL-OLD is a 24-item self-report instrument that is divided into six Facets: Facet 1- Sensory Abilities (SA); Facet 2- Autonomy (A); Facet 3- Past, Present, and Future Activities (PPFA); Facet 4- Social Participation (SP); Facet 5- Death and Dying (DD); and Facet 6- Intimacy (I) (4 items per subscale). Each facet provides an individual score, and an overall score (general QOL – GQOL) is also calculated from the set of 24 items. Answers are based on a 5-point Likert response scale, with items 1, 2, 6, 7, 8, 9, 10 being reverse scored. Although all the response scales have five points they vary in their anchors: “Not at all”/“An extreme amount”; “Completely”/“Extremely”; “Very poor”/“Very good”; “Very dissatisfied”/“Very satisfied”; “Very unhappy”/“Very happy”). Total scores on the WHOQOL-OLD range from 4 to 20, with higher scores being indicative of better quality of life (QOL).

All the test were administered in and indoor sports center, under the same environmental conditions for each participant.

#### MHRCT protocol

Initially, prior to the commencement of the study, the subjects were submitted to two weeks of MHRCT, two sessions per week, in order to familiarize with the MHRCT exercises performed in the current study. During this familiarization period a higher emphasis was placed on learning the proper exercise techniques and brief pauses between repetitions were allowed in order to reset their starting positions when necessary^49^. In the second week, participants were also measured for body mass, height, fat mass, and quality of life questionnaires. In the same session, elderly completed the submaximal strength test 1-RM loads for chest press (CHP) and leg extension (LE), and the next day, completed the military press (MP), and hip extension (HE), and then, after 72 hours, the 1RM tests were repeated to determine test-retest reliability. In these testing sessions, participants were also familiarized with the OMNI-RES scale^46,47^.

A supervised progressive MHRCT program designed to induce muscular hypertrophy was performed. EG followed a progressive MHRCT program for 12-weeks. The CG not participated in the MHRCT program. The training program incorporated resistance exercise of six major regions and consisted of 3 training sessions per week on non-consecutive days (Monday, Wednesday and Friday).

All subjects performed the sets with moderate-intensity (8 to 12 repetitions) in each exercise. The load was increased during the 12 weeks from 60% 1-RM^22,24^ to high-intensity 80% 1-RM^24,32,50^. The training load was increased when the individual could perform more than the prescribed number of repetitions (12 repetitions) followed the OMNI-RES scale^46,47^ and a hard effort perception level. Rest between sets was 1–2 minutes.

All training sessions were monitored by a physical education professional expert and the subjects were not allowed to perform another exercises program during the training period.

### Statistical analysis

The statistical program SPSS v21.0 (Chicago, IL) was used for the data analysis and processing. Preliminary analyses included the testing of assumptions such as normality, homogeneity of variances. No violations in data normality were evident from the Kolmogorov-Smirnov test, which led to the use of parametric statistics. Pre-test scores were therefore included as covariates in subsequent analysis of variables^51^.

In the quality of life variables, Cronbach’s alpha coefficient was employed to calculate the internal reliability for each questionnaire item (facet 1: **α** = 0.73; facet 2: **α** = 0.81; facet 3: **α** = 0.78; facet 4: **α** = 0.71; facet 5: **α** = 0.76; facet 6: **α** = 0.80) and global score (**α** = 0.77).

Likewise, all variables were analysed according to ANCOVA to examine if there were statistically significant differences in this type of variable between two groups. Pre-test scores there was statistically significant differences was included as covariable. In the ANCOVA, variation of each variable based on time were calculated as Δ = Post intervention data – Pre intervention data.

For all variables, the level of statistical significance was established at *p* ≤ 0.05, with a confidence interval for differences of 95%. Effect sizes were calculated using the partial eta-squared statistic (ηp2) to establish the substantive meaningfulness of the differences found^52^.

## Results

### Preliminary analysis

At baseline, as an average, the subjects inclueded in both sex also in both group, according standard categorizations of World Health Organization were identified as overweight (BMI: 25.0–29.9 kg/m^2^). With a mean (SD) BMI women CG of 26.1 (3.3) kg/m^2^, BMI women EG of 27.6 (4.4) kg/m^2^, BMI men CG of 26.7 (4.5) kg/m^2^ and BMI men EG of 27.9 (3.7) kg/m^2^. Subject characteristics are presented in Table 1. Significant between-group differences were observed in diferent variables in women and men. Regarding GDLAM, women and men showed baseline diferences between groups at SSP. About strength conditioning, women presented diferences in 1RM CHP (kg), and men diferences were in 1RM LE (kg) and 1RM HE. Regarding Quality of life questionnaire, Women and men showed differences in “facet 6”, but just in case of women presented differences at questionnaire total score as well. However, we controlled for potential confounders between the two groups using age, BMI, and uneven baseline scores of measures as covariates in the statistical analysis.

### Body composition outcomes

**Figure 2:**
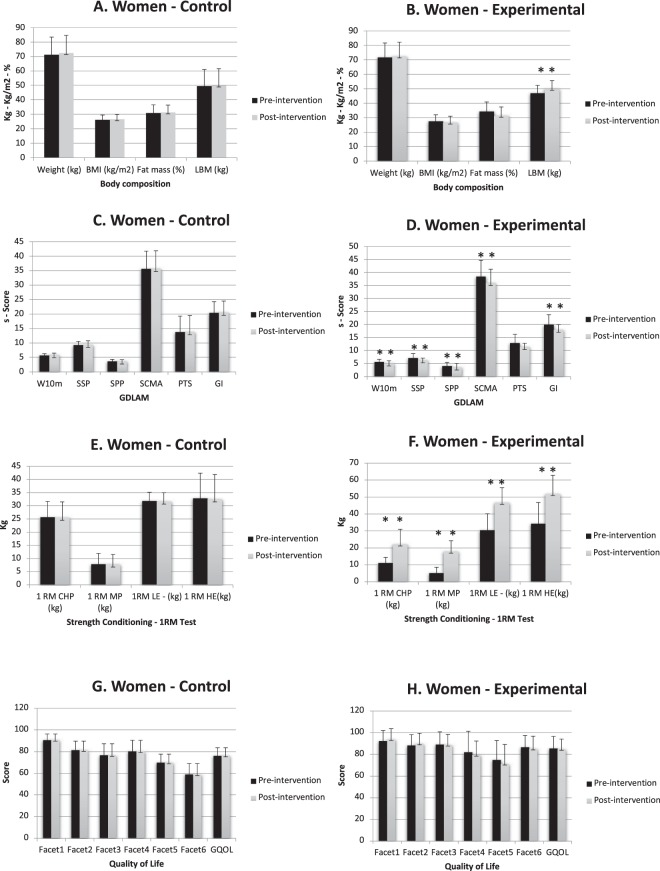
Women pre-intervention and post-intervention comparison. *pValue < 0.05; **pValue < 0.01; BMI: Body Mass Index; LBM: Lean Body Mass; GDLAM: Latin American Group for Maturity protocol; W10m: walking 10 m test; SSP: standing up from a seated position test; SPP: standing up from a prone position; SCMA: standing up from a chair and moving about the room; PTS: putting on and taking off a shirt; GI: GDLAM index; RM: 1-Repetitium maximum; CHP: Chest Press; MP: Military Press; LE: Leg Extension; HE: Hip Extension; QOL: Quality Of Life.

Women: Fig. 2A,B show the body composition variables from CG and EG, respectively, at the begging or end of the study for women. In the EG, LBM (kg) was significantly higher (p < 0.010) post-intervention. No observed changes in body composition in the CG. Table 2 presents the adjusted mean change in the body composition, were EG (95% CI: 0.73–2.54; p < 0.05) increased LBM in comparison with CG (95% CI: 0.73–1.61; p < 0.05).

**Table 2:** ANCOVA Women comparison control group vs experimental group.

Body composition	Control (n = 12)		Experimental (n = 15)		Mean dif.	F (gl)	p Value	Eta^2^	OP	R^2^
	X	SD	X	SD						
ΔWeight (kg)	1.27	0.55	−0.01	1.50	0.5357	2.193	0.074	0.563	0.713	0.306
Δ BMI (kg/m^2^)	0.47	0.21	0.00	0.59	0.205	2.193	0.074	0.563	0.713	0.306
Δ Fat mass (%)	0.42	0.69	−2.14	1.88	−1.0464	2.576	0.127	0.132	0.328	0.786
Δ LBM (kg)	0.46	1.18	1.62	0.79	1.1214	3.333	**0.014**	0.662	0.902	0.464
**GDLAM**										
Δ W10m	0.20	0.13	−0.49	0.41	−0.1929	4.93	**0.002**	0.744	0.984	0.593
Δ SSP	0.13	0.21	−0.82	1.49	−0.4143	6.143	**0.024**	0.265	0.647	0.597
Δ SPP	0.12	0.36	0.06	1.69	0.0821	2.759	**0.032**	0.619	0.826	0.394
Δ SCMA	0.13	0.64	−2.45	3.05	−1.3464	5.034	**0.002**	0.748	0.986	0.599
Δ PTS	0.12	0.17	−1.44	2.97	−0.775	2.151	0.079	0.559	0.703	0.299
ΔGI	0.20	0.27	−1.95	2.43	−0.959	9.487	**0.005**	0.283	0.84	0.253
**Strength conditioning**										
Δ 1 RM CHP (kg)	−0.11	0.70	11.11	7.02	6.3	1.36	0.260	0.074	0.196	0.601
Δ 1 RM MP (kg)	−0.11	0.45	12.70	5.98	7.2107	11.34	**<0.001**	0.87	1	0.793
Δ 1RM LE - (kg)	−0.19	0.28	16.26	7.54	9.2107	27.868	**<0.001**	0.943	1	0.909
Δ 1 RM HE(kg)	−0.41	0.39	17.54	14.14	9.8464	7.773	**<0.001**	0.821	1	0.715
**Quality of Life**										
Δ Facet 1	0.14	0.22	1.56	14.70	0.8929	0.86	0.584	0.336	0.295	−0.055
Δ Facet 2	0.20	0.17	1.56	10.08	0.8929	0.841	0.598	0.331	0.288	−0.062
Δ Facet 3	−0.11	0.21	−0.39	11.29	−0.2232	0.898	0.357	0.05	0.146	0.073
Δ Facet 4	0.18	0.34	−2.60	14.58	−1.4882	1.55	0.205	0.477	0.531	0.169
Δ Facet 5	−0.23	0.13	−3.78	19.99	−2.1575	1.301	0.305	0.433	0.448	0.1
Δ Facet 6	0.37	0.24	−1.56	6.65	−0.8929	0.584	0.455	0.033	0.111	−0.018
Δ Global QOL	0.21	0.19	−0.77	9.78	−0.4404	3.706	0.071	0.179	0.443	0.04

Data expressed as difference between the variable at the beginning and end of the study; Δ = Post intervention data – Pre intervention data; M: Mean; SD: Standard Deviation; Mean dif: Mean difference; OP: Observed power; BMI: Body Mass Index; LBM: Lean Body Mass; GDLAM: Latin American Group for Maturity protocol; W10m: walking 10 m test; SSP: standing up from a seated position test; SPP: standing up from a prone position; SCMA: standing up from a chair and moving about the room; PTS: putting on and taking off a shirt; GI: GDLAM index; RM: 1-Repetitium maximum; CHP: Chest Press; MP: Military Press; LE: Leg Extension; HE: Hip Extension; QOL: Quality Of Life.

**Figure 3:**
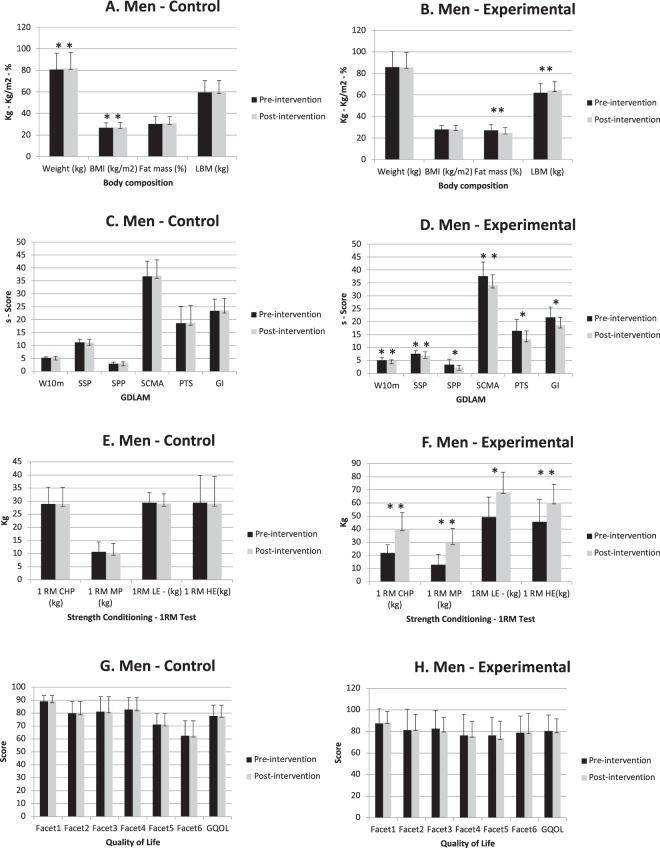
Men pre-intervention and post-intervention comparison. *pValue < 0.05; **pValue < 0.01; BMI: Body Mass Index; LBM: Lean Body Mass; GDLAM: Latin American Group for Maturity protocol; W10 m: walking 10 m test; SSP: standing up from a seated position test; SPP: standing up from a prone position; SCMA: standing up from a chair and moving about the room; PTS: putting on and taking off a shirt; GI: GDLAM index; RM: 1-Repetitium maximum; CHP: Chest Press; MP: Military Press; LE: Leg Extension; HE: Hip Extension; QOL: Quality Of Life.

Men: Intra-group comparison showed significant differences in both CG (Fig. 3A) and EG (Fig. 3B). CG presents an increment of total body weight (p < 0.01) and BMI (p < 0.01) at the end of the study. However, EG maintain weight and BMI, and shows higher values of LBM (p < 0.01) and lower of fat mass (p < 0.01) after intervention. Also compared with CG in the Table 3, EG presented significantly decrease in weight (95% CI CG: −1.11–0.63; EG: 0.19–1.76; p < 0.05) fat mass (95% CI CG: −1.50–1.31; EG: −3.16–0.624; p < 0.05) and BMI (95% CI CG: −0.36–0.21; EG: 0.07–0.58; p < 0.05), but an increase of LBM (95% CI CG: −0.88–0.87; EG: 1.26–2.85; p < 0.01).

**Table 3:** ANCOVA Men comparison control group vs experimental group.

Body composition	Control (n = 9)		Experimental (n = 9)		Mean dif	F	p Value	Eta^2^	OP	R^2^
	X	DS	X	DS						
Weight (kg)	0.95	0.57	−0.09	0.87	0.40	4.44	**0.041**	0.88	0.73	0.68
BMI (kg/m^2^)	0.31	0.19	−0.02	0.28	0.14	4.36	**0.042**	0.88	0.72	0.68
Fat mass (%)	0.39	1.06	−2.32	0.88	−1.05	6.23	**0.018**	0.91	0.87	0.77
LBM (kg)	0.04	1.34	2.02	0.75	1.09	12.43	**0.003**	0.95	0.99	0.88
**GDLAM**										
W10m	0.16	0.20	−0.43	0.63	−0.15	14.42	**0.002**	0.96	1.00	0.89
SSP	−0.13	0.16	−0.79	1.18	−0.48	0.52	0.500	0.08	0.09	0.19
SPP	0.20	0.19	−1.13	1.54	−0.51	6.28	**0.018**	0.91	0.87	0.77
SCMA	0.19	0.75	−3.58	2.37	−1.81	6.92	**0.014**	0.92	0.91	0.79
PTS	0.21	0.16	−3.26	1.93	−1.62	5.80	**0.022**	0.91	0.84	0.75
GI	0.21	0.19	−3.11	1.50	−1.54	37.96	**0.000**	0.72	1.00	0.70
**Strength conditioning**										
1 RM CHP (kg)	0.04	0.88	18.06	9.48	9.58	3.57	0.066	0.86	0.63	0.62
1 RM MP (kg)	−0.36	0.40	16.57	7.92	8.60	6.00	**0.020**	0.91	0.86	0.76
1RM LE - (kg)	−0.31	0.34	19.07	19.24	9.95	22.01	**0.003**	0.79	0.97	0.82
1 RM HE(kg)	−0.30	0.47	14.68	12.15	7.63	2.90	0.139	0.33	0.30	0.68
**Quality of Life**										
Facet 1	0.18	0.20	0.93	10.42	0.49	3.65	0.063	0.86	0.64	0.62
Facet 2	0.11	0.15	0.69	14.80	0.37	0.43	0.886	0.42	0.11	−0.55
Facet 3	0.13	0.30	−2.08	15.31	−1.10	0.76	0.664	0.56	0.16	−0.17
Facet 4	−0.20	0.19	−0.69	14.13	−0.37	0.86	0.603	0.59	0.17	−0.09
Facet 5	−0.22	0.23	−2.78	15.02	−1.47	0.26	0.969	0.31	0.08	−0.85
Facet 6	0.15	0.14	0.46	6.14	0.25	1.50	0.266	0.20	0.18	0.12
Global QOL	0.15	0.17	−0.48	9.79	−0.25	0.43	0.883	0.42	0.11	−0.55

Data expressed as difference between the variable at the beginning and end of the study; M: Mean; SD: Standar Deviation; Mean dif: Mean difference; OP: Observed power; BMI: Body Mass Index; LBM: Lean Body Mass; GDLAM: Latin American Group for Maturity protocol; W10m: walking 10 m test; SSP: standing up from a seated position test; SPP: standing up from a prone position; SCMA: standing up from a chair and moving about the room; PTS: putting on and taking off a shirt; GI: GDLAM index; RM: 1-Repetitium maximum; CHP: Chest Press; MP: Military Press; LE: Leg Extension; HE: Hip Extension; QOL: Quality Of Life.

### Functional autonomy outcomes

Women: Fig. 2C,D presents GDLAM test protocol divided by group CG and EG respectively. EG showed significantly decrease in all GDLAM test (all test p < 0.01) except at PTS test (no difference). No observed differences at CG. Similar results were observed for GDLAM test when CG and EG were compared (Table 2). No differences between groups were presented at PTS test. Nevertheless, significant differences were observed in the W10m (95% CI CG: −0.42–0.44; EG: −0.68–0.01; p < 0.01), SSP (95% CI CG: 0.26–1.96; EG: −2.11–0.79; p < 0.05), SPP (95% CI CG: −0.44–2.41; EG: −1.70–0.50; p < 0.05), SCMA (95% CI CG: −1.27–3.52; EG: −5.05–1.35; p < 0.01), and GI (95% CI CG: −2.91–2.71; EG: −1.79–2.74; p < 0.01).

Men: Significant changes pre and post intervention were reported in all GDLAM protocol tests in the EG (Fig. 3D). However, CG did not show any differences after intervention (Fig. 3C). Table 3 shows the comparison between groups, and presented differences in all GDLAM test, W10m (95% CI CG: −0.2–0.42; EG: −0.67–0.11; p < 0.01), SPP (95% CI CG: −1.66–0.83; EG: −1.4–0.85; p < 0.05), SCMA (95% CI CG: −1.12–3.04; EG: −6.14–2.38; p < 0.01), PTS (95% CI CG: −1.49–1.32; EG: −2.09–0.45; p < 0.05), and GI. (95% CI CG: −0.42–4.30; EG: −6.92–3.41; p < 0.01). Except at SSP test, were differences between groups not founded.

### Muscular strength outcomes

Women: Relative to the baseline and the end of the study, no changes were observed in strength conditioning variables in the CG (Fig. 2E). EG (Fig. 2F) shows significant differences in all RM test: CHP, MP, LE and HE (all p < 0.01). Significant between-group differences were also obtained at Table 2, where EG showed higher values in almost all test: MP (95% CI CG: −7.6–2.56; EG: 10.58–18.47; p < 0.01), LE (95% CI CG: 0.87–9.53; EG: 8.87–15.56; p < 0.01), HE (95% CI CG: −11,51–9.71; EG: 9.71–26.10; p < 0.01). RM-CHP did not present a significant difference.

Men: CG participants did no present changes in strength variables at the end of the study in comparison with the base line (Fig. 3E). In contrast, EG showed significant increase at these all variables or test (Fig. 3F): CHP (p < 0.01), MP (p < 0.01), RM-LE (p < 0.05) and HE (p < 0.01). In addition, after group comparison, EG always shows higher values than CG (Table 3). Significant between-group differences were founded in the RM test: CHP (95% CI CG: −10–83–13.69; EG: 5.79–27.89; p < 0.05), MP (95% CI CG: −9.41–8.50; EG: 8.56–24.49; p < 0.05), LE (95% CI CG: −12.87–11.86; EG: 8.07–30.40; p < 0.05) test. But no differences were observed at RM-CHP.

### Quality of life outcomes

Figure 2G,H show punctuation marks in each facet and a global score for women participants. Figure 3G,H show the same variables for men. None significant between-group (Tables 2 and 3) or timeline differences were obtained, in women either in men about quality of life variables.

## Discussion

In the present study we investigated the effects of a progressive MHRCT on body composition, FA, MS and QOL on healthy elderly. As hypothesized, positive changes were observed in body composition, also in FA and MS in both gender, nevertheless, changes in QOL perception were not observerd, after 12 weeks of a progressive MHRCT.

As other authors show in their research^50,53^, this study highlights the advantages of applying a MHRCT program on body composition, physical function, all with the aim to prevent and delay muscle weakness, as a potential factor in maintaining independence of older people and thus try to predispose this population to healthy aging.

In our study, no modification in women of BMI because BMI is an imprecise term to determine changes after intervention in body composition^54^. Also the problem that there are no variations or changes in BMI can also be due to a more effective range is needed to determine and/or define the standard rhythms of corporal composition; or rather, nutritional status in this population, as discussed findings from other studies^7,55,56^.

However, if there are changes in the percentage of fat mass and LBM in men EG can attribute these changes as benefits as associated with the effects of MHRCT, because have not experienced such changes in the CG, as other previus studies which done similar resistance intervention and body composition assessment^57–60^. In women, only changes on LBM were observed, similar than other studies^61^, and it could be related on women after menopause have an increased abdominal fat mass, and to show an effective reduction of fat mass must combine resistance training plus caloric restriction^62^.

Some authors suggest a combination of diet and regular exercise to modulate the reduction of functional capacity relative to age, delaying the onset or progression of functional disability^63^. Thus, a good dietary approach, along with regular physical exercise, has been associated with a lower risk of chronic diseases (coronary, obesity, diabetes, sarcopenia, osteoporosis, etc.)^14,64^. In this sense, the effects of the combination of isocaloric diet plus RT in older people can result in an increase in muscle mass^65,66^. These factors could justify those found in the present study in which EG had an improvement in LBM and percentage of fat, but did not obtain changes in BMI.

The results of the assessments of MS found in this study showed a significant improvement for upper and lower extremities in the group that did the MHRCT in men and women (except in chest press RM test). This is corroborated by other authors, which performed resistance program training in elderly^57,59,67–69^. As Vale *et al*.^3^, to argue that a systematic program of physical exercise can increase muscle strength in elderly people, causing enhanced capabilities to perform their activities of daily living^70^. With the performance of their motor functions developed, the elderly can increase the life expectancy of healthy and active way, especially with the independence and autonomy^62^.

Clemson *et al*.^70^ applied an exercise intervention for balance and lower limb strength for 12 months in order to prevent falls. The authors found increases in muscle strength by dynamometry and reduction in the time of execution of the TUG balance test. Activities of daily living improved by the Physical Activity Scale for the Elderly, showed small but significant gains for the LiFE program.

Other study shows that, low intensity RT 40 to 50% of 1RM, was effective in increasing MS and reducing total fat mass^22,33,71^. However, RT with higher 1-RM percentages provides muscle strength gains in a faster period of time^4,26,29^. In the present study, in addition to these results obtained by the EG, the exercises proposed in the study intervention meet the needs of MS exercises for the elderly^72^.

The results of this study corroborate those reported int the literature about the enhancement of FA after resistance program intervention in elderly^68,69^. Our results show that the FA of older people has improved significantly in women and men, in almost all the EG test, not having improvements in CG. In addition, the program showed improvement in the index of autonomy GDLAM (IG) in just 12 weeks of intervention. It is improved functional abilities of the elderly, which offers independence in old age. This may mean more independence to perform activities of daily life, thus improving self-esteem and their perception of the quality of life of the elderly^41,42^.

The results of QOL for this study showed that EG were not for facets of the WHOQOL-OLD questionnaire^35^. In terms of effects on QOL, showed that QOL perception of EG group did not present significant improvement in comparison to the CG in men or women population. Probably no differences in this variable may due to low sample or because the intervention was performed for 12 weeks. For future studies, researchers must take into consideration to include a re-test some months after the training intervention, due to training adaptation could report more QOL benefits in long term. The results of the present study corroborate those reported in the literature for periodized RT programs, because they can be effective in body composition, muscular strength and FA^53,71^. Other types of intervention have also shown increases in MS, FA and QOL^62,73–78^.

Therefore, the progressive MHRCT can be an excellent non-pharmacological method for controlling the negative effects of aging on the control aspects of % fat mass, FA and promoting independence, maintenance or improvement of MS and therefore a healthier life in the elderly, who may even reduce health care costs.

Mariano *et al*.^79^ performed an intervention twice a week, 60 minutes in duration, for 12 weeks. The intensity was established by the zone of maximum repetitions (three to four series, 8 to 12 repetitions) and the order of the exercises was modified every four weeks. The experimental group achieved significant strength gain scores in knee extensors (p = 0.0032, 30.23%) and lumbar spine extensors (p = 0.0207, 12.33%). The QOL evaluation was significant, with a percentage increase in the FA capacity domains (p = 0.0092, 11.05%), general health status (p = 0.0075, 14.17%), vitality (P = 0.0015, 15.38%) and mental health (p = 0.0154, 9.64%). Thus, RT promoted a significant increase in MS, affecting the improvement of QOL in the domains of functional capacity, general health, vitality and mental health. These results also occurred in the present study with the application of a progressive MHRCT. Thus, it can be observed that progressive MHRCT can be an efficient strategy to improve the general health of the elderly. In addition, a recent study^80^, shows the importance of coaches motivating intrinsically older people towards RT programmes. The authors propose 10 motivational strategies to apply in the RT programmes. This will achieve adherence and physiological and psychosocial benefits in the MHRCT elderly practitioner. Regular practice of physical exercise is fundamental for the maintenance of health, functional independence, and QOL in old age; however, the dropout rates of programs offering this type of activity are high. In the present study, the number of dropouts were because older people might also be more likely to interrupt their exercise programs because the incidence of chronic diseases increases with advancing age^81^, as we can see in Fig. 1. Other reasons for the older adults to abandon physical exercise were lack of time, emergence of diseases, and occurrence of illness in relatives^82^. Further research into more effective forms of MHRCT and combining RT and aerobic exercise and motivational RT programs are needed. A limitation of this study are related to the purposive selection of the sample, which is not the gold standard for generalization of information.

## Conclusions

This study showed that a 12-week progressive MHRCT (from 60% 1-RM to 80% 1-RM) designed in the present study is associated with significant increase in upper and lower muscular strength as well as functional capacity and significantly improvements body composition and physical in elderly.

Older people need more information about the health (physical, psychological and social) benefits of doing RT. Our study indicates that this progressive MHRCT program should be promoted for the elderly as it has the potential to improve physical performance, thereby prolonging healthy independent aging, but the quality of life perception probably needs more weeks of intervention to cause changes. Progressive MHRCT with moderate to high loads should be incorporated in training and readaptation programs of ageing and frail older adults under the direction by a physical education professional.
